# Non-overweight depressed patients who respond to antidepressant treatment have a higher risk of later metabolic syndrome: findings from the METADAP cohort

**DOI:** 10.1017/S0033291722003919

**Published:** 2023-10

**Authors:** K. El Asmar, N. B. Annan, R. Khoury, R. Colle, S. Martin, T. E. Ghoul, S. Trabado, P. Chanson, B. Feve, C. Verstuyft, L. Becquemont, E. Corruble

**Affiliations:** 1 CESP, MOODS Team, INSERM UMR 1018, Faculté de Médecine, Univ. Paris-Saclay, Le Kremlin Bicêtre F-94275, France; 2 Department of Epidemiology and Population Health, Faculty of Health Sciences, American University of Beirut, Beirut, Lebanon; 3 Service Hospitalo-Universitaire de Psychiatrie de Bicêtre, Hôpitaux Universitaires Paris-Saclay, Assistance Publique Hôpitaux de Paris, Hôpital de Bicêtre, Le Kremlin Bicêtre F-94275, France; 4 INSERM UMR-S U1185, Faculté de Médecine, Univ. Paris-Saclay, Le Kremlin Bicêtre F-94275, France; 5 Service de Génétique Moléculaire, Pharmacogénétique et Hormonologie de Bicêtre, Hôpitaux Universitaires Paris-Saclay, Assistance Publique-Hôpitaux de Paris, Hôpital de Bicêtre, Le Kremlin Bicêtre F-94275, France; 6 Service d'Endocrinologie et des Maladies de la Reproduction, Centre de Référence des Maladies Rares de l'Hypophyse, Assistance Publique-Hôpitaux de Paris, Hôpital de Bicêtre, Le Kremlin Bicêtre F-94275, France; 7 Sorbonne Université-INSERM, Centre de Recherche Saint-Antoine, Institut Hospitalo-Universitaire ICAN, Service d'Endocrinologie, Hôpital Saint-Antoine, Assistance Publique-Hôpitaux de Paris, Paris F-75012, France; 8 Centre de recherche clinique, Hôpitaux Universitaires Paris-Saclay, Assistance Publique-Hôpitaux de Paris, Hôpital de Bicêtre, Le Kremlin Bicêtre F-94275, France

**Keywords:** Antidepressant, depression, MetS, response to treatment, weight

## Abstract

**Background:**

Major depressive disorder (MDD) is a complex disorder with a significant public health burden. Depression remission is often associated with weight gain, a major risk factor for metabolic syndrome (MetS). The primary objective of our study was to assess prospectively the impact of response to antidepressant treatment on developing MetS in a sample of MDD patients with a current major depressive episode (MDE) and who are newly initiating their treatment.

**Methods:**

In the 6-month prospective METADAP cohort, non-overweight patients, body mass index <25 kg/m^2^, with MDD and a current MDE were assessed for treatment response after 3 months of treatment, and incidence of MetS after 3 and 6 months of treatment. Outcome variables were MetS, number of MetS criteria, and each MetS criterion (high waist circumference, high blood pressure, high triglyceridemia, low high-density lipoprotein-cholesterolemia, and high fasting plasma glucose).

**Results:**

In total, 98/169 patients (58%) responded to treatment after 3 months. A total of 2.7% (1/38) developed MetS out of which 12.7% (10/79) (*p* value < 0.001) had responded to treatment after 3 months. The fixed-effect regression models showed that those who responded to treatment after 3 months of follow-up had an 8.6 times higher odds of developing MetS (odds ratio = 8.58, 95% confidence interval 3.89–18.93, *p* value < 0.001).

**Conclusion:**

Compared to non-responders, non-overweight patients who responded to treatment after 3 months of antidepressant treatment had a significantly higher risk of developing MetS during the 6 months of treatment. Psychiatrists and nurses should closely monitor the metabolic profile of their patients, especially those who respond to treatment.

## Introduction

Major depressive disorder (MDD) is a heterogeneous and complex disorder with a significant public health burden. It confers a 50% increased mortality of somatic causes (Vos et al., 2012), especially from metabolic syndrome (MetS) (Pan et al., 2012; Pulkki-Råback et al., 2009; Sekita et al., 2013; Vancampfort et al., 2014). In addition to its direct burden, MDD is also a major contributor to cardiovascular diseases (CVDs), which rank first in terms of global disease burden. Vigo, Thornicroft, and Atun (2016) have estimated that the global burden for mental illness accounts for 32.4% of years of healthy life lost due to disability and 13.0% of disability-adjusted life years.

MetS is a cluster of five risk factors for CVDs and type 2 diabetes mellitus. It includes high waist circumference (WC), high triglyceridemia (TG), high blood pressure (BP), high fasting plasma glucose (FPG), and low high-density lipoprotein cholesterolemia (HDL-C). It is established that the use of antidepressants and antipsychotics can lead to weight gain among individuals who suffer from depression, bipolar disorder, or schizophrenia (Delacrétaz et al., 2017; Salvi, Mencacci, & Barone-Adesi, 2016b). In turn, weight gain was also prospectively associated with onset of MetS. The METADAP cohort reported that the incidence of MetS among in-patients with MDD and without MetS at baseline was 11.7% and 16.5% after 3 and 6 months, respectively (Corruble et al., 2015). Another publication from the same cohort, linked early weight gain (after 30 days of antidepressant treatment) to later incidence of MetS [odds ratio (OR) 5.5] and a higher number of MetS criteria Incidence Rate Ratio ((IRR) = 1.7). Early weight gain (as little as 3% in the first 30 days) was also found to predict later weight gain (>15% after 6 months follow-up) (El Asmar et al., 2018). Weight gain is therefore an established risk factor for MetS and can therefore be looked and considered as a proxy for MetS in such a population. There is a paucity of evidence examining the relationship between response or remission after antidepressant treatment and weight gain, let alone the relationship between response/remission and MetS.

Response to antidepressants is linked to genetic, environmental factors, and genetic × environment interactions. While the association between resistance to antidepressants and existing MetS among patients was reported in the literature (Anderson, Gott, Sayuk, Freedland, & Lustman, 2010; Cattaneo et al., 2013), only one study reported a strong correlation between response to antipsychotics, not antidepressants, and the onset of metabolic dysregulations among those who have schizophrenia (Hönig, 2018). And even though depression remission was often associated with weight gain, it was not clear if weight gain was an effect of recovery from depression or the result of the pharmacological effect of antidepressants (Fava, 2000; Russ & Ackerman, 1988). For example, Benazzi (1998) has found, based on 100 unipolar/bipolar remitted depressed patients, that 72% of this sample have gained weight compared to their weight during major depression.

To our knowledge, no study has investigated the prospective relationship between response to treatment (RTT) and MetS. However, due to the increased mortality associated with obesity and the high burden of CVDs associated with MetS, prevention of MetS becomes a major issue for MDD patients. Therefore, the primary objective of our study was to assess prospectively the impact of response to AD treatment on developing MetS in a sample of MDD patients with a current major depressive episode (MDE) and who have initiated new antidepressant treatment.

## Methods

### Design

The data are part of METADAP, a 6-month prospective, multi-centric, real-world treatment study (NCT00526383) (Corruble et al., 2015). It assessed MetS at baseline and post-antidepressant treatment in MDD patients with a current MDE. Data were collected between June 2008 and March 2013 from six university psychiatry departments in France. MDE patients were enrolled and assessed at the beginning of an index antidepressant prescription, as well as 1, 3, and 6 months later.

### Patients

Our study sample included in and outpatients, aged between 18 and 65, with research-confirmed diagnosis of current MDE in a context of MDD based on the Mini International Neuropsychiatric Interview (MINI 5.0), with a minimum depression score of 18 on the 17-item Hamilton Depression Rating Scale (HDRS) (Hamilton, 1960) and requiring the beginning of a new or switching from another antidepressant treatment. Patients included had a body mass index <25 kg/m^2^ and did not have MetS at baseline.

We excluded patients with psychotic symptoms, bipolar disorders, psychotic disorders, eating disorders, current substance abuse or dependence (DSM-IV-TR), pregnancy, breastfeeding, organic brain syndromes, or severe unstable medical conditions. Patients taking antipsychotics or mood stabilizers were also not included. We also excluded patients with medical conditions associated with risk for weight gain, such as diabetes, hyperthyroidism, and polycystic ovary syndrome.

Written informed consent was obtained was from study participants. Interviews and diagnostic assignments were reviewed by a senior psychiatrist, independently from the treating psychiatrist. The index antidepressant treatment had to belong to one of the four following classes: selective serotonin reuptake inhibitors (SSRIs), serotonin norepinephrine reuptake inhibitors (SNRIs), Tricyclics (TCAs), or other antidepressant treatments (others). A monotherapy of antidepressants was required. If antidepressant treatment was changed, patients were dropped out of the study.

### Assessment of RTT

RTT was measured at M3 and M6 using the Hamilton Rating Scale for Depression (HDRS) scale: a 50% decrease from baseline HDRS score indicated that the patient responded to treatment (Rush et al., 2006).

### Assessment of MetS and parameters

Before initiation of antidepressant treatment, patients' baseline metabolic measures were assessed. MetS and its individual metabolic dysregulations (WC, TG, HDL, FPG, DBP, SBP) were assessed at three, and six months (M3, M6, respectively) of Antidepressant (AD) treatment. MetS was defined using the definition of the International Federation of Diabetes (IFD) (Alberti, Zimmet, & Shaw, 2006), and was considered as dichotomous variable (present/absent). Training and quality control were performed to ensure that all measures were systematically identified and collected at all visits.

### Measures

Additionally, 5% weight gain after 30 days of treatment initiation was calculated, along with the duration of depression (in years) and the class of AD. An increase in appetite after 3 months of treatment was reported using the clinician rating of the Quick Inventory of Depressive Symptomatology (QIDS-C) (Rush et al., 2003), dosage of antidepressant drugs was considered as low, normal and high, depending on the recommended daily dosage and the maximum tolerated dose for every drug (online Supplementary Table S1).

### Statistical analysis

The primary outcome variable was MetS incidence. Secondary outcomes were the number of MetS dysregulations (range 0–5) and the 5 individual parameters: high WC, high FPG, high BP, low HDL-C and high TG. RTT at 3 months was the primary exposure variable.

Pearson χ^2^ tests and independent sample *t* tests were used to assess statistical difference in clinical and sociodemographic characteristics with respect to RTT at M3 (Table 1). The associations between the metabolic parameters at baseline, 3, and 6 months follow-up, and RTT at M3 were tested using univariate logistic regression adjusted for center effect. Similarly, subgroup analysis was completed on those who completed the 6 months follow-up (Table 2). Mixed-effect logit and Poisson models were used to test the longitudinal association between RTT and the metabolic variables.

**Table 1.** Comparison between those who responded to treatment at M1 and M3 for socio-demographics and metabolic variables^a^

	Total			M3				
				RTT		NRTT		
	*N* = 169	%	(Test statistics) *p*^b^	*N* = 98	%	*N* = 71	%	(Test statistic) p*
Age, *m* (s.d.)	42.0 (13.2)			40.9 (13.1)		43.6 (13.4)		(*t* = 1.32, df = 167) 0.19
Sex								
Females	114/169	67.5		64/98	65.3	50/71	70.4	(χ^2^ = 0.49, df = 1) 0.48
MDD duration (years), *m* (s.d.)	9.7 (11.9)			8.3 (10.8)		11.7 (13.1)		(*t* = 1.81, df = 167) 0.07
HDRS score at baseline, *m* (s.d.)	25.1 (4.9)			25.7 (5.1)		24.2 (4.5)		(*t* = −2.07, df = 167) 0.04
5% weight gain at M1	29/169	17.2		17/98	17.4	12/71	16.9	(χ^2^ = 0.01, df = 1) 0.94
Class of AD								
SSRI	77/169	45.6		51/98	52.0	26/71	36.6	(χ^2^ = 9.42, df = 3) 0.02
SNRI	54/169	32.0		33/98	33.7	21/71	29.6	
TCA	12/169	7.1		4/98	4.1	8/71	11.3	
Other	26/169	15.3		10/98	10.2	16/71	22.5	
Dose at baseline								
Low	13/159	8.2		4/97	4.1	9/62	14.5	(χ^2^ = 5.60, df = 2) 0.05
Normal	144/159	90.6		92/97	94.8	52/62	83.9	
High	2/159	1.2		1/97	1.1	1/62	1.6	
MetS^b^								
M0, *n*/*N*	0	0	*Z* = 3.31 <0.01	0	0	0	0	
M3, *n*/*N*	15/167	9.0		11/98	11.2	4/69	5.8	(*Z* = 1.19) 0.24
M6, *n*/*N*	12/120	10.0		10/79	12.7	1/38	2.6	(*Z* = 7.93) <0.01
No. of MetS dysregulations^b^								
M0, *m* (s.d.)	1.0 (0.8)		*Z* = 2.57 0.01	1.0 (0.8)		1.1 (0.9)		(*Z* = −1.71) 0.09
M3, *m* (s.d.)	1.2 (1.0)			1.1 (1.1)		1.2 (0.9)		(*Z* = −0.17) 0.87
M6, *m* (s.d.)	1.4 (1.1)			1.3 (1.2)		1.4 (1.0)		(*Z* = −0.63) 0.53
High WC^b^								
M0, *n*/*N*	66/169	39.1	*Z* = 4.01 <0.01	34/98	34.7	32/71	45.1	(*Z* = −3.06) 0.001
M3, *n*/*N*	79/168	47.0		40/98	40.8	39/70	55.7	(*Z* = −4.64) <0.001
M6, *n*/*N*	67/120	55.8		42/80	52.5	25/40	62.5	(*Z* = −1.56) 0.12
High FPG^b^								
M0, *n*/*N*	13/168	7.7	*Z* = 2.18 0.03	8/97	8.3	5/71	7.0	(*Z* = 1.15) 0.25
M3, *n*/*N*	27/168	16.1		20/98	20.4	7/70	10.0	(*Z* = 1.66) 0.10
M6, *n*/*N*	18/119	15.1		14/79	17.7	4/40	10.0	(*Z* = 2.42) 0.02
High BP^b^								
M0, *n*/*N*	28/169	16.6	*Z* = 3.14 <0.01	15/98	15.3	13/71	18.3	(*Z* = −0.49) 0.62
M3, *n*/*N*	43/169	25.4		28/98	28.6	15/71	21.1	(*Z* = 1.37) 0.17
M6, *n*/*N*	36/120	30.0		27/80	33.8	9/40	22.5	(*Z* = 2.33) 0.02
High TG^b^								
M0, *n*/*N*	19/169	11.2	*Z* = 2.03 0.04	11/98	11.2	8/71	11.3	(*Z* = −0.01) 0.99
M3, *n*/*N*	22/169	13.0		14/98	14.3	8/71	11.3	(*Z* = 2.01) 0.04
M6, *n*/*N*	20/120	16.7		14/80	17.5	6/40	15.0	(*Z* = 0.42) 0.68
Low HDL^b^								
M0, *n*/*N*	47/169	27.8	*Z* = −2.42 0.02	28/98	28.6	19/71	26.8	(*Z* = 0.30) 0.76
M3, *n*/*N*	24/168	14.3		10/97	10.3	14/71	19.7	(*Z* = −2.65) <0.01
M6, *n*/*N*	22/120	18.3		9/80	11.3	13/40	32.5	(*Z* = −3.56) <0.01

a *p values were calculated using χ^2^ tests or Fisher exact test (dosage) for categorical variables and independent sample *t* test for continuous variable. *p* values for the metabolic parameters were calculated using univariate logistic regression adjusting for center effect.

b *p* values reported from logistic regression mixed models accounting for the repeated measures of the metabolic variables over the 6 months follow-up.

**Table 2.** Comparison between those who responded to treatment and those who did not respond to treatment at M3 for socio-demographic and metabolic variables in the subgroup of completers^a^

	Total completers			RTT			NRTT			
	*N* = 121	%	*p*^b^	81	66.9%	*p*^b^	40	33.1%	*p*^b^	(Test statistics) p*
Age, *m* (s.d.)	42.3 (13.0)			42.5 (13.4)			42.1 (12.3)			(*t* = −0.16, df = 119) 0.87
Sex										
Females	83	68.6		52	64.2		31	77.5		(χ^2^ = 2.19, df = 1) 0.14
MDD duration (years), *m* (s.d.)	9.7 (11.6)			9.1 (11.3)			10.9 (12.4)			(*t* = 0.76, df = 119) 0.45
HDRS score at baseline, *m* (s.d.)	24.9 (5.0)			25.7 (5.2)			23.3 (4.3)			(*t* = −2.5, df = 119) 0.01
5% weight gain at M1	16	13.2		13	16.1		3	7.5		(χ^2^ = 1.71, df = 1) 0.19
Change in appetite at M3										
No change	62/121	51.2		48/81	59.3		14/40	35		(χ^2^ = 16.15, df = 2) <0.01
Increase	31/121	25.6		23/81	28.4		8/40	20		
Decrease	28/121	23.1		10/81	12.3		18/40	45		
Class of AD, *n*										
SSRI	55/121	45.5		40/81	49.4		15/40	37.5		(χ^2^ = 6.31, df = 3) 0.10
SNRI	42/121	34.7		30/81	37.0		12/40	30.0		
TCA	10/121	8.3		4/81	4.9		6/40	15.0		
Other	14/121	11.6		7/81	8.6		7/40	17.5		
Dose										
Low	10	8.5		3/81	3.7		7/37	18.9		(χ^2^ = 8.04, df = 2) 0.01
Normal	106	89.8		77/81	95.1		29/37	78.4		
High	2	1.7		1/81	1.2		1/37	2.7		
MetS										
M0, *n*/*N*	0/121	0	<0.01	0/81	0	<0.01	0/40	0	0.36	
M3, *n*/*N*	11/121	9.1		9/81	11.1		2/40	5.0		(*Z* = 1.14) 0.25
M6, *n*/*N*	11/117	9.4		10/79	12.7		1/38	2.6		(*Z* = 7.93) <0.01
No. of MetS dysregulations										
M0, *m* (s.d.)	1.0 (0.84)		0.02	0.9 (0.8)		<0.04	1.1 (0.9)		0.27	(*Z* = −2.2) 0.03
M3, *m* (s.d.)	1.2 (1.0)			1.1 (1.1)			1.2 (0.9)			(*Z* = −0.46) 0.65
M6, *m* (s.d.)	1.4 (1.1)			1.3 (1.2)			1.4 (1.0)			(*Z* = −0.63) 0.53
High WC										
M0, *n*/*N*	49/121	40.5	<0.01	28/81	34.6	<0.01	21/40	52.5	0.12	(*Z* = −2.66) <0.01
M3, *n*/*N*	57/121	47.1		33/81	40.7		24/40	60.0		(*Z* = −5.49) <0.01
M6, *n*/*N*	67/120	55.8		42/80	52.5		25/40	62.5		(*Z* = −1.56) 0.12
High FPG										
M0, *n*/*N*	10/120	8.3	0.05	8/80	10.0	0.07	2/40	5.0	0.39	(*Z* = 4.21) <0.01
M3, *n*/*N*	20/121	16.5		16/81	19.8		4/40	10.0		(*Z* = 2.50) 0.01
M6, *n*/*N*	18/119	15.1		14/79	17.7		4/40	10.0		(*Z* = 2.42) 0.02
High BP										
M0, *n*/*N*	18/121	14.9	0.01	13/81	16.1	<0.01	5/40	12.5	0.17	(*Z* = 0.47) 0.64
M3, *n*/*N*	33/121	27.3		25/81	30.9		8/40	20.0		(*Z* = 2.00) 0.05
M6, *n*/*N*	36/120	30.0		27/80	33.8		9/40	22.5		(*Z* = 2.33) 0.02
High TG										
M0, *n*/*N*	13/121	10.7	0.05	8/81	9.9	0.06	5/40	12.5	0.56	(*Z* = −0.73) 0.47
M3, *n*/*N*	15/121	12.4		10/81	12.4		5/40	12.5		(*Z* = −0.08) 0.94
M6, *n*/*N*	20/120	16.7		14/80	17.5		6/40	15.0		(*Z* = 0.42) 0.68
Low HDL										
M0, *n*/*N*	34/121	28.1	0.02	21/81	25.9	<0.01	13/40	32.5	1.000	(*Z* = −0.83) 0.41
M3, *n*/*N*	14/120	11.7		7/80	8.8		7/40	17.5		(*Z* = −2.24) 0.03
M6, *n*/*N*	22/120	18.3		9/80	11.3		13/40	32.5		(*Z* = −3.56) <0.01

a *p values were calculated using χ^2^ tests or Fisher exact test (dosage) for categorical variables and independent sample *t* test for continuous variable. *p* values for the metabolic parameters were calculated using univariate logistic regression adjusting for center effect.

b *p* values reported from logistic and Poisson regression mixed models accounting for the repeated measures of the metabolic parameters over the 6 months follow-up.

The adjusted associations between the outcome and exposure variables were measured using logistic regression models accounting for center effect. All regression models were adjusted for age (in years), sex, lifetime duration of MDD (in years), severity of depression measured using HDRS scores at baseline, antidepressant dosage at baseline, increase in appetite after 3 months, and 5% weight gain at M1. A two-tailed alpha level of 5% was used to indicate statistical significance. All analyses were performed using STATA 13 MP.

## Results

### Sample characteristics

**Fig. 1.**
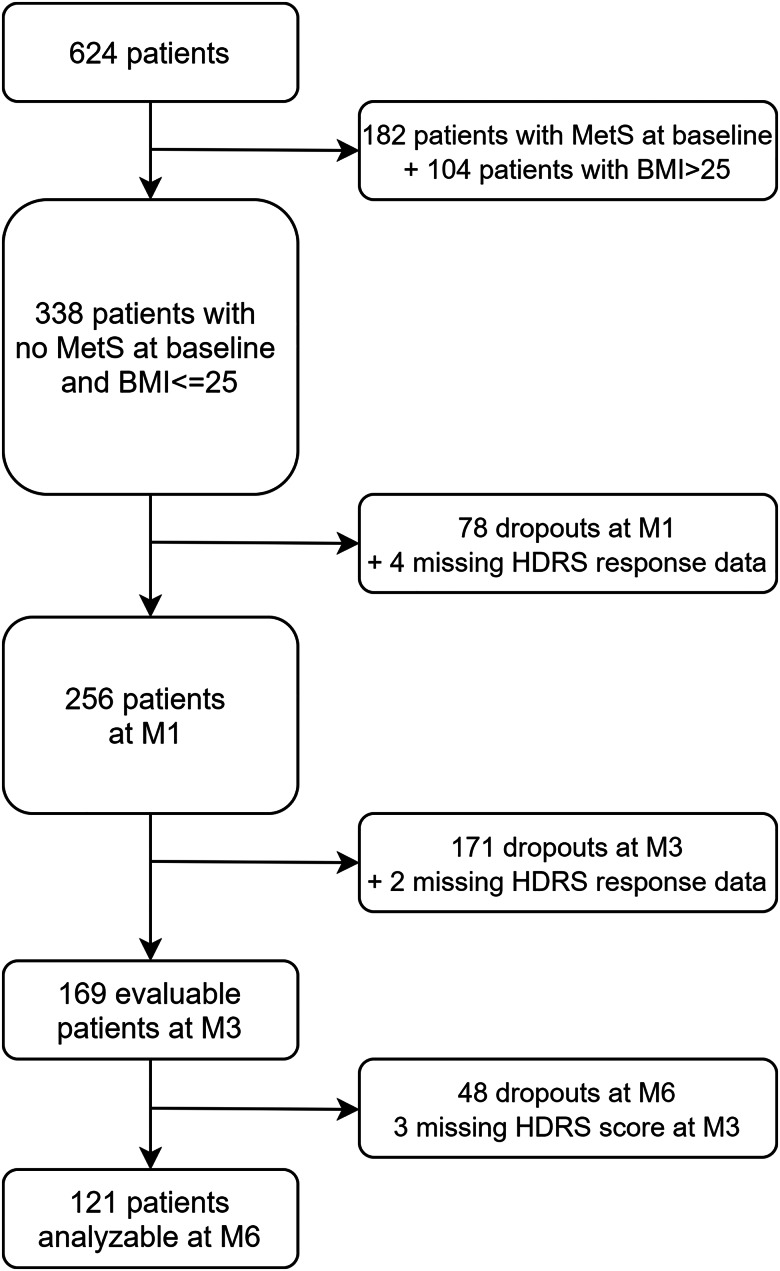
Patients’ flowchart.

Our sample consisted of 169 patients, of whom 114 (67.5%) were females, and their mean age was 42.0 years (s.d. 13.2) (Fig. 1). The average duration of MDD was 9.7 years (s.d. 11.9), and the average HDRS score at baseline was 25.1 (± 4.9) (Table 1). At the time of enrollment in the study, most of the patients (45.6%) were being treated with SSRIs, followed by SNRIs (32.0%), TCAs (7.1%), and others (15.3%). Before their enrollment in the study, 33/169 patients (19.5%), 95/169 (56.2%), and 53/169 (31.4%) had already been prescribed TCAs, SNRIs, and SSRIs, respectively, as a previous AD treatment. In total, 14/33 (42.4%) patients on TCAs before their enrollment in the study switched to either SSRI or SNRI, and only 9/111 (8.1%) patients who were on either SSRI or SNRI have shifted to TCA. Out of the 14 participants who shifted from TCA to either SSRI or SNRI, 9 (64.3%) responded to treatment after 3 months of follow-up. After 30 days of treatment, 29/169 participants (17.2%) had 5% weight gain. In the completer's subgroup, 28.4% (23/81) of patients who RTT reported an increase in their appetite compared to 20% (8/40) in the no RTT group (NRTT) (*p* value < 0.001) (Table 2).

Most of the patients (90.6%) were prescribed a standard dose of antidepressant. Within the completers' subset, the proportion of patients being prescribed a low dose of AD at baseline was higher among the NRTT group (*p* value = 0.01). The total number of patients who responded to treatment (RTT) after 3 months was 98/169 (58%), while those who did not respond (NRTT) were 71/169 (42%) (Table 1).

The two response groups (RTT and NRTT) did not differ on age (*p* = 0.19) and sex (*p* = 0.48). Mean HDRS score at baseline was significantly higher in the RTT group after 3 months: 25.7 (s.d. 5.1) *v.* 24.2 (s.d. 4.5) (*p* = 0.04) (Table 1). Among those who completed the treatment, 67% in the RTT (*n* = 81, 67%) had a significantly higher baseline HDRS score compared to the NRTT group. The difference in HDRS score at 3 months was also significant: 25.7 (s.d. 5.2) *v.* 23.3 (s.d. 4.3) (*p* = 0.01) (Table 2).

Completers and non-completers did not differ in age, sex, lifetime duration of MDD, lifetime duration of prior antidepressant medication, HDRS score at baseline, or current antidepressant treatment. Among the 124 patients completing the 6-month follow-up period, three had missing HDRS scores at the third month follow-up visit, and four had missing data on at least one of the five MetS criteria, so MetS incidence after 6 months could only be reported for 117 patients (Table 2).

### MetS and metabolic parameters

In the whole sample, all MetS parameters (high WC, high BP, low HDL, high TG, high FPG) significantly worsened over the 6-month follow-up period. MetS incidence increased to 9% (15/167) and 10% (12/120) after 3 and 6 months, respectively (*p* < 0.01). The average number of MetS dysregulations per patient increased from 1.0 to 1.4 in the whole sample (*p* = 0.01) (Table 1). Similar trends were observed among completers, except for high FPG (*p* = 0.05).

### RTT as a predictor of MetS and MetS dysregulations

Patients in the RTT group after 3 months had a higher incidence of MetS after 6 months [12.7% (10/79) *v.* 2.6% (1/38), *p* < 0.01].

Results from the mixed-effect logistic and Poisson models showed that there was no significant change in any of the metabolic variables in the NRTT strata (Table 2). Within the RTT group, MetS incidence increased to 12.7% after 6 months of follow-up (*p* < 0.01). Similarly, the average number of MetS dysregulations increased from 0.8 to 1.2 (*p* = 0.04). Over the 6-month follow-up period, the incidence of high WC, and high BP increased from 34.6% to 52.5% (*p* < 0.01), and 16.1% to 33.8% (*p* < 0.01), respectively. Interestingly, the incidence of low HDL decreased from 25.9% to 11.3% (*p* < 0.01).

At baseline, the prevalence of high WC was significantly higher in the NRTT group [45.1% (32/71) *v.* 34.7% (34/98), *p* < 0.01], but no significant difference could be found at 6 months follow-up. The incidence of high FPG increased after 6 months of follow-up from 8.3% to 17.7% in the RTT group and from 7% to only 10% in the NRTT group; the difference at 6 months was statistically significant between the two response groups (*p* = 0.02). The incidence of high BP after 6 months was higher in the RTT group, rising from 15.3% at baseline to 33.8% compared to 18.3% at baseline and 22.5% for the NRTT group. The difference was statistically significant between the two groups at 6 months (*p* = 0.02) (Table 1).

In the completers' subgroup, similar trends of MetS and high FPG incidence were observed as in the total sample. In addition, the incidence of high BP was higher in the RTT group at 3 months [30.9% (25/81) *v.* 20% (8/40), *p* value = 0.045] and at 6 months [33.8% (27/80) *v.* 22.5% (9/40), *p* value = 0.020]. The incidence of high WC was significantly higher at baseline and at 3 months but not at 6 months in the NRTT compared to the RTT group (52.5% *v.* 34.6%, *p* value = 0.002 at baseline, 40.7% *v.* 60.0%, *p* < 0.01 at 3 months, 62.5% *v.* 52.5%, *p* = 0.12 at 6 months). However, results from the stratified mixed-effect logistic models show that the increase in the incidence of high WC was only statistically significant in the RTT subgroup (*p* < 0.02 *v. p* = 0.13). The incidence of low HDL was higher in the NRTT group at baseline, 3, and 6 months (32.5% *v.* 25.9%, *p* = 0.41 at baseline, 17.5% *v.* 8.8%, *p* = 0.03 at 3 months, 32.5% *v.* 11.3%, *p* value < 0.01 at 6 months) (Table 2).

The fixed-effect regression models (Table 3) showed that after 3 months the RTT group had an 8.6 times higher odds of developing MetS [OR 8.6, 95% confidence interval (CI) 3.9–18.9, *p* < 0.01], and three times higher odds of high FPG incidence (OR 3.1, 95% CI 1.6–6.0, *p* = 0.01) after 6 months follow-up. Consistently with the results from the stratified analysis, those who responded to AD treatment, were at five times lower odds of having low HDL levels (OR 0.2, 95% CI 0.1–0.4, *p* < 0.01). These associations are independent from age, sex, HDRS score at baseline, MDD duration, early weight gain, increase in appetite, class and dosage of antidepressant.

**Table 3.** Multivariate logistic regression for the RTT at M3 as a predictor of later incident of MetS and each parameter at M6^a^

	Risk measure (OR)	95% CI	*p*
MetS	8.6	3.9–18.9	<0.01
No. of MetS dysregulations	0.9	0.6–1.3	0.47
High WC	0.8	0.3–2.0	0.59
High FPG	3.1	1.6–6.0	<0.01
High BP	1.7	0.7–4.1	0.28
High TG	0.6	0.2–2.1	0.47
Low HDL-C	0.2	0.1–0.4	<0.01

a [*N* = 117 (four patients had missing data on at least one metabolic parameter)]. Results controlling for age, sex, HDRS score at M0, MDD duration, 5% weight gain at M1, class of AD treatment, dosage, and increase in appetite.

**Fig. 2.**
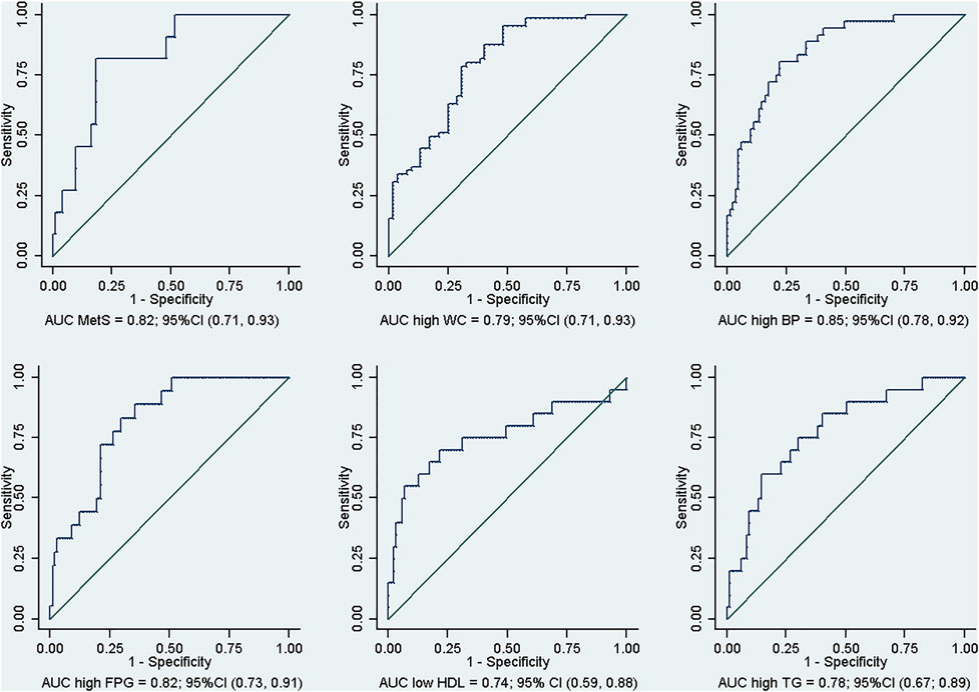
Receiver operating curve of RTT after 3 months of follow-up, for MetS and each of its dysregulation.

RTT had good discriminatory power for all MetS-related variables, with an area under the receiver operating curve (AUC) averaging to 0.8 for all models (Fig. 2).

## Discussion

Among depressed patients with normal weight and no MetS, RTT after 3 months of initiation predicts the later onset of MetS and several other metabolic dysregulations. Patients in the RTT group had almost nine times higher odds of developing MetS, compared to those in the NRTT group.

The incidence of MetS is considerably higher among patients in the RTT group, with the odds of MetS incidence being around 8.6 times higher compared to those in the NRTT group. Patients who responded to treatment were also at an elevated risk of high FPG incidence. Interestingly, low HDL was associated with NRTT. The variation of the metabolic parameters as well as the overall MetS incidence was restricted to the RTT group. The prediction power of RTT is considered robust, with an average AUC of 0.8 for the six models. Our analysis controlled for major confounding variables such as severity of depression at baseline, early weight gain, class of antidepressant treatment, increase in appetite, and drug dosage.

To the best of our knowledge, no other study has explored the impact of response to antidepressant treatment and the onset of MetS in a population of depressed adults with normal weight and no MetS. Some studies have investigated the impact between MetS and lack of response or diminished response to antidepressant or antipsychotic treatment (Dale, Bryant, & Thompson, 2020; Sagud et al., 2013; Strawbridge, Young, & Cleare, 2018), but never in the opposite direction.

The incidence of MetS at 6 months was significantly greater among responders. This difference in incidence is mainly driven by the higher incidence of high FPG and high BP at 6 months among the RTT group. Patients who responded to treatment had a significant increase in their WC compared to those who did not respond, this could be due to patients regaining their appetite, which has subsequently led to weight gain. Our results showed that the proportion of patients who reported an increased appetite after 3 months of follow-up is greater than among those of RTT. This may explain the more pronounced increase in WC in this group, with the proportion of patients presenting high WC increasing from 34.6% at baseline to 52.5% after 6 months of follow-up. Weight gain has also been previously associated with depression remission (Fava, 2000) as well as MetS in an adult population suffering from MDD and being treated with AD (El Asmar et al., 2018).

HDL was the only metabolic parameter that improved in the RTT group, with the proportion of patients presenting this specific dysregulation dropping from 25.9% at baseline to 11.3% after 6 months follow-up. Fixed-effect regression models have also shown that RTT was associated with an improvement in this specific metabolic parameter (OR 0.19, *p* value < 0.001). We believe that since the observed metabolic dysregulations are mostly weight gain driven and since low HDL-C is less sensitive to weight gain compared to FPG and hypertension, it improved due to the recovery from depression. A previous publication on the same cohort of patients has shown that low HDL-C was the least sensitive metabolic parameter to early weight gain (Asmar et al., 2018).

Higher incidence of MetS among RTT group could be explained by the different types of antidepressant drug treatments used by the patient; those displaying high antihistaminergic activity may significantly contribute to the onset of MetS in MDD patients (Salvi, Barone-Adesi, D'Ambrosio, Albert, & Maina, 2016a). This was also supported by other findings; blocking of hypothalamic H1 receptors by second-generation antipsychotics may also contribute to fat accumulation (high WC) by decreasing lipolysis in some tissues and increasing lipogenesis in white adipose tissue (He, Deng, & Huang, 2013). Therefore, in order to prevent the onset of MetS, clinicians should consider the medication's pharmacodynamics profile rather than just class when determining which type of treatment is suitable for a certain patient. Also, TCAs negatively impact BP because they influence the heart's vagal control. SSRIs may be the best choice for treating depression or anxiety disorders, particularly in individuals with comorbid CVD. TCA treatment in these patients should be combined with rigorous BP monitoring if these fail to demonstrate therapeutic efficacy (Licht et al., 2009). Remission from depression can increase appetite, causing fat accumulation and an increase in WC. In turn, these factors could lead to the onset of MetS.

There are several strengths that could be underlined in the present study. It is the first prospective real-life cohort to assess prospectively the impact of response to antidepressant treatment on MetS and its dysregulations on MDE patients treated with AD monotherapy, with normal weight, and without MetS. Our analysis also considers major socio-demographic, therapeutic, and clinical characteristics, including lifetime duration of depression, severity of depression at baseline, and dosage and class of the current AD treatment. Another strength lies in our multivariate analysis, which showed that the association between RTT on the one hand and MetS, high FPG, and low HDL-C remain significant after controlling for several demographic and clinical variables such as chronicity (duration of depression).

As chronically depressed patients who failed to respond to SSRI and SNRI treatments tend to shift toward TCAs, the trends in METADAP were different (Bauer et al., 2008; Chockalingam, Gott, & Conway, 2019). For 42% of METADAP patients who were prescribed TCAs prior to their enrollment in the study have switched to either SSRI or SNRI, while only 8.1% have shifted from SSRI and SNRI to TCA. Furthermore, 64% of those shifting to SSRI and SNRI have responded to treatment after 3 months. This supports the hypothesis that in our sample, chronicity and resistance to treatment were not necessarily linked with TCAs, which are known to be associated with higher metabolic adverse events.

Findings from the stratified analysis (by RTT), reinforce our findings that RTT is associated with MetS, by showing that only the RTT group had an increased risk of MetS incidence and dysregulations in other metabolic parameters. Furthermore, our findings are based on a particularly pure sample of clinically depressed patients with no MetS and who are non-overweight. With no studies published on this specific subset of patients, our findings are considered highly relevant as they provide an insight about the pathway connecting AD monotherapy with subsequent RTT and later MetS incidence.

There are some limitations to our study. First, the analyzed sample is not to be considered a representative sample of the general French MDD population as it was collected from six university psychiatric departments. Second, our study protocol did not take into consideration the change in dietary and physical activity patterns, despite available evidence of the benefits of such lifestyle modification (Yamaoka & Tango, 2012). Third, because medication selection was not randomly assigned, the treating physician may have considered the possibility of depression resistance – based on the history of the patient – when prescribing the new treatment. Fourth, due to the absence of weight-related data prior to depression, it was not possible to ascertain whether the observed weight gain was a mere resumption of the previous weight that was due to the possible regain on appetite. Fifth, the dropout rate of this sample was relatively high, and the results from the multivariate analysis were based on 120 out of the 169 included patients; however, the dropout rate in our sample was quite similar to that of STAR-D (Warden et al., 2009a, 2009b). Attrition had to be looked at taking RTT into consideration. In fact, non-response is an undesired outcome which could lead to dropout. In our study, 43% of NRTT group have discontinued as opposed to 17.4% in the RTT group (*p* value < 0.001). The attrition rate/drug discontinuation among the NRTT group is consistent with other prospective studies from the literature on treating depression (Bajbouj et al., 2010; Sirey et al., 2020). Furthermore, if treatment discontinuation could be a result of both NRTT and metabolic abnormalities, then the high attrition rate would have induced an underestimation of our effect size. Thus, it can be expected that a lower attrition rate would have increased the magnitude of the reported association.

## Conclusion

Response to antidepressant treatment after 3 months of treatment initiation significantly increases the odds of developing MetS after 6 months. The question of whether the increased incidence in MetS among patients who respond to antidepressant treatment is a pharmacological and/or a recovery effect would still be legitimate. Psychiatrists and nurses are advised to closely monitor the metabolic profile of their patients, especially those who respond to treatment. Specifically, they should monitor patient's WC and FPG in the first 6 months of treatment.

## Supplementary material

For supplementary material accompanying this paper visit https://doi.org/10.1017/S0033291722003919. click here to view supplementary material
