# Supplementary material for: Non-overweight depressed patients who respond to antidepressant treatment have a higher risk of later metabolic syndrome: findings from the METADAP cohort
Source: Psychol Med. 2023 Jan 11;53(14):6560–9. doi: 10.1017/S0033291722003919 (PMC10600935; doi:10.1017/S0033291722003919)
Supplement: Supplementary file 1 [file S0033291722003919sup001.docx]

| **Molecules** | **Normal dose** |
| --- | --- |
| Anafranil / Clomipramine | 75-150 mg |
| Deroxat / Paroxétine (chlorydrate) | 20-50 mg |
| Floxyfral / Fluvoxamine | 100-300 mg |
| Prozac / Fluoxétine | 20-60 mg |
| Séroplex / Escitalopram | 10-20 mg |
| Séropram / Citalopram | 20-40 mg |
| Zoloft / Sertraline | 50-200 mg |
| Cymbalta / Duloxétine | 60-120 |
| Effexor / Venlafaxine | 75-375 |
| Norset / Mirtazapine | 15-45 |
| Marsilid / Iproniazide | 25-50 |
| Moclamine / Moclobémide | 300-600 |
| Laroxyl / Amitriptyline | 75-150 |
| Ludiomil / Maprotiline | 75-150 |

**Supplemental table 1: Threshold values used to categorized drugs into dosages: low, normal and high**
